# Cholesterol-Lowering Potentials of Lactic Acid Bacteria Based on Bile-Salt Hydrolase Activity and Effect of Potent Strains on Cholesterol Metabolism *In Vitro* and *In Vivo*

**DOI:** 10.1155/2014/690752

**Published:** 2014-11-03

**Authors:** Cheng-Chih Tsai, Pei-Pei Lin, You-Miin Hsieh, Zi-yi Zhang, Hui-Ching Wu, Chun-Chih Huang

**Affiliations:** ^1^Department of Food Science and Technology, Hungkuang University, No. 1018, Section 6, Taiwan Boulevard, Shalu District, Taichung City 43302, Taiwan; ^2^Graduate Institute of Clinical Medical Science, China Medical University, Taichung City 40402, Taiwan; ^3^Department of Food and Nutrition, Providence University, Taichung City 43301, Taiwan

## Abstract

This study collected different probiotic isolates from animal and plant sources to evaluate the bile-salt hydrolase activity of probiotics *in vitro*. The deconjugation potential of bile acid was determined using high-performance liquid chromatography. HepG2 cells were cultured with probiotic strains with high BSH activity. The triglyceride (TG) and apolipoprotein B (apo B) secretion by HepG2 cells were evaluated. Our results show that the BSH activity and bile-acid deconjugation abilities of *Pediococcus acidilactici* NBHK002, *Bifidobacterium adolescentis* NBHK006, *Lactobacillus rhamnosus* NBHK007, and *Lactobacillus acidophilus* NBHK008 were higher than those of the other probiotic strains. The cholesterol concentration in cholesterol micelles was reduced within 24 h. NBHK007 reduced the TG secretion by 100% after 48 h of incubation. NBHK002, NBHK006, and NBHK007 could reduce apo B secretion by 33%, 38%, and 39%, respectively, after 24 h of incubation. The product PROBIO S-23 produced a greater decrease in the total concentration of cholesterol, low-density lipoprotein, TG, and thiobarbituric acid reactive substance in the serum or livers of hamsters with hypercholesterolemia compared with that of hamsters fed with a high-fat and high-cholesterol diet. These results show that the three probiotic strains of lactic acid bacteria are better candidates for reducing the risk of cardiovascular disease.

## 1. Introduction

Cholesterol is a vital substance in the human body. Long-standing elevated levels of blood cholesterol may lead to atherosclerosis and may therefore pose a major risk for developing cardiovascular diseases (CVDs). The World Health Organization (WHO) reported that CVDs were responsible for 30% of deaths worldwide and predicted that CVDs will remain the leading causes of death in the coming two decades. By the year 2030, CVDs will affect approximately 23.3 million people around the world [1]. WHO also reported that a 10% reduction in serum cholesterol in men aged 40 could decrease the incidence of heart disease within 5 years by 50% [1]. Both drug therapy and nonpharmacologic approaches, including dietary intervention, behaviour modification, and regular exercise, are common strategies to lower blood cholesterol levels [2]. Despite the proven cholesterol-lowering ability of certain pharmacological agents, unwanted side effects can occur in some cases, such as gastrointestinal discomfort [3].

Probiotics are defined by the Food and Agriculture Organization (FAO) and WHO as living microorganisms which when administered in adequate amounts confer upon the host a health benefit [4]. In the 1970s fermented milk containing a wild* Lactobacillus* strain was reported to have a hypocholesterolemic effect in humans [5]. Since then, many experiments have been conducted* in vitro* or* in vivo* to investigate the cholesterol-lowering effect of lactic acid bacteria (LAB), especially strains of* Lactobacillus* and* Bifidobacterium* [6–8]. In a review by Pereira and Gibson [9] of studies on the hypocholesterolemic effect of probiotics, they concluded that dairy products fermented with the appropriate strain(s) of bacteria might induce a decrease in the level of circulating cholesterol concentrations. However, the strains found in fermented dairy products do not normally reside in the human intestinal tract [9]. Thus, daily consumption of probiotic products may be a dietary solution for inducing long-term hypocholesterolemic effects.

Several mechanisms for cholesterol removal by probiotics have been proposed, such as deconjugation of bile salts by bile-salt hydrolase (BSH) [10], assimilation of cholesterol into bacterial cell membranes [11], production of short-chain fatty acids (SCFAs) during the growth of probiotics [12], and cholesterol conversion into coprostanol [13]. Nondeconjugating organisms do not appear to have the ability to remove cholesterol from the culture medium to a significant extent. In contrast, lactobacilli with BSH activity have the ability to survive and colonize the lower small intestine where the enterohepatic cycle takes place. Therefore, BSH activity is considered an important colonization factor and an essential criterion for the selection of probiotic isolates with cholesterol-lowering properties [14].

Based on the ability of certain probiotic lactobacilli and bifidobacteria to deconjugate bile acids enzymatically, Sanders [15] proposed that the BSH activity mechanism increases the rate of excretion. Such mechanism could be used in controlling serum cholesterol levels by colonic microbes. In the present study we identified and characterized strains of LAB with BSH activity and evaluated its potential* in vitro* and* in vivo* as a cholesterol-reducing probiotic. Our objective was to develop a new LAB product that could serve as a probiotic that reduces cholesterol levels in humans.

## 2. Materials and Methods

### 2.1. Bacterial Strains, Culture Medium, and Growth Conditions

Eight hundred LAB strains obtained from faeces of healthy infants, from plant sources, or from the Bioresource Collection and Research Center (BCRC; Hsinchu, Taiwan) were screened. Each stock culture was maintained in 20% glycerol at −80°C. Bacterial cells were propagated twice in lactobacilli Man-Rogosa-Sharpe (MRS) broth (DIFCO, Detroit, Michigan, USA) with 0.05% L-cysteine and incubated at 37°C for 20 h. The cells were centrifuged (10,000 g for 10 min at 4°C) to obtain a 20-hour-old spent culture supernatant (SCS) with cell density adjusted to (1–9) × 10^9^ CFU/mL.

### 2.2. Screening of Cultures for BSH Activity

Isolates were initially selected on the basis of Gram reaction, morphology, and catalase activity. All Gram-positive and catalase-negative rods were selected to determine BSH activity. Sterile filter discs (6 mm) were impregnated with an overnight culture and then placed on MRS agar plates supplemented with 0.5% (w/v) sodium salt of taurodeoxycholic acid or taurocholic acid (TCA) (Sigma, St. Louis, MO, USA) and 0.37 g/L CaCl_2_ [16]. Plates were incubated anaerobically at 37°C for 72 h, after which the diameter of the precipitation zones was measured.

### 2.3. Quantitative BSH Activity

Sterile MRS broth (50 mL) was supplemented with a filter-sterilised solution of 1.0 mmol/L TCA (Sigma, St. Louis, MO, USA) before use. LAB strains were inoculated (1%, v/v) in MRS broth and grown under anaerobic conditions at 37°C for 24 h. Samples were taken aseptically at various time intervals (0, 4, 8, 12, and 24 h) during incubation. The optical density of each sample was measured at 600 nm to monitor cell growth. The concentration and pH of bile acids was also determined. Each experiment was performed in triplicate for each strain and uninoculated MRS broth supplemented with TCA was used as a control. The BSH enzymatic activity of deconjugated TCA compared with that of the control was expressed as a percentage [16].

A modification of the high-performance liquid chromatography (HPLC) method described by de Smet et al. [17] was used to quantitatively determine the BSH activity. A reversed-phase column (Gemini C18; 5 *μ*m; 110 Å; 250 × 4.6 mm) (Phenomenex, Aschaffenburg, Germany) was used. Free and conjugated bile acids were eluted under a linear gradient using methanol in aqueous buffer at a flow rate of 1.0 mL/min. Mobile phases were 0.3% ammonium carbonate (solvent A), 100% acetonitrile (solvent B), and HPLC-grade methanol (5% and 65%; solvents C and D, resp.). The gradient elution program used was as follows: isocratic elution performed with 27% solvent B and 73% solvent A for 10 min followed by 10 min linear gradient to 32% solvent B and 68% solvent A. The mobile-phase composition was finally maintained at 50% solvent B and 50% solvent A for 10 min. The detection wavelength was set at 210 nm and chromatography was performed at room temperature. The injection volume was 20 *μ*L. TCA (Sigma, 97% purity) was used as a standard.

### 2.4. Bile-Salt Extracts

Cells were separated from the solution by centrifugation (8,000 g for 10 min at 5°C) to remove bile salts from the MRS broth cultures. A modification of the method described by de Smet et al. [17] was used to recover bile salts from the SCS. The supernatant (1 mL) was acidified by addition of 10 *μ*L of 6 N HCl to stop BSH activity. Lithocholic acid was used as an internal standard and was added to a final concentration of 8 mmol/L. Isopropanol (4 mL) was used to extract the bile salts (1 : 4, v/v). The samples were mixed for 60 min at 420 rpm and then centrifuged at 1,000 g for 10 min. The isopropanol layer was transferred to a clean test tube and then evaporated under N_2_ flow at 37°C. After complete isopropanol removal the bile-salt extract was redissolved in 800 *μ*L of methanol and then filtered through a 0.45 *μ*m HPLC filter (Millipore, Bedford, MA, USA). Prior to injection into the HPLC filter, the samples were stored at −20°C.

### 2.5. Measurement of Cholesterol in Mixed Micelles

A modified method was developed from the technique described by Raederstorff et al. [18] and Gilliland et al. [19]. Mixed micelles were prepared by sonication (130 W, 20 kHz) of the MRS medium containing 6.6 mmol/L TCA, 2.4 mmol/L lecithin, and 0.5 mmol/L cholesterol. The lipids were dissolved in methanol and then dried before the MRS medium was added. The micellar dispersion was filtered through a sterile 0.45 *μ*m filter (Millipore, Bedford, MA, USA) and then stored at 4°C for 48 h.

The 1% LAB suspension was inoculated into 2.5 mL of the micellar dispersion and the resulting dispersion was then incubated for 18 h at 37°C. A sample of the micellar dispersion (1 mL) was taken at different time points (0, 6, 12, 18, and 24 h) and centrifuged at 7000 g for 10 min. The samples (0.5 mL) were transferred to clean test tubes. Ethanol (3 mL, 95%) and KOH (2 mL, 50%) were then added sequentially to each tube before thorough mixing and heating for 10 min at 60°C in a water bath. The samples were cooled and 5 mL of hexane was dispensed into each tube. Mixing for 30 sec was done, followed by repeated washing with 3 mL of distilled water. The tubes were allowed to stand for 15 min at room temperature for phase separation. The hexane layer (2.5 mL) was transferred into a clean test tube and then evaporated at 60°C using a stream of nitrogen gas. To each tube was added 4 mL of *ο*-phthalaldehyde reagent (Sigma, St. Louis, MO, USA). The tubes were allowed to stand at room temperature for 10 min and then 2 mL of concentrated sulphuric acid was pipetted slowly down the wall of each tube. The contents of each tube were immediately mixed. After the test tubes were stored at room temperature for an additional 10 min, the absorbance at 550 nm was read against a reagent blank. The cholesterol concentration (1 mmol/L) (99% standard for chromatography; Sigma) was determined from the absorbance at 550 nm using a standard curve. Results were expressed as micrograms of cholesterol per millilitre.

### 2.6. Cell Culture and Secretion of Apolipoprotein B (Apo B) and TG

A monolayer of the HepG2 cell line (BCRC 60025) was obtained from BCRC. The cells were maintained in Eagle's minimum essential medium with 10% foetal bovine serum and 50 U/mL penicillin-streptomycin solution (Gibco, Grand Island, NY) at 37°C and 5% CO_2_. HepG2 cells were subcultured in 10 cm dishes (Corning Costar) to 80% confluence. Since HepG2 cells are highly dependent on a high concentration of exogenous fatty acids to maintain an adequate supply of lipids for lipoprotein assembly [20], we investigated the LAB-SCS effect on apo B and triglyceride (TG) secretion in cells incubated with oleic acid (OA). OA was provided as a complex with bovine serum albumin (BSA). The OA/BSA molar ratio was 8 : 1, and the concentrations were 0.81 mmol/L for OA and 0.1 mmol/L for BSA [21, 22].

HepG2 cells (10^5^ cell/mL) were incubated in 24-well plates (Corning Costar) with or without 100 *μ*L LAB-SCS per well overnight in an incubator (37°C, 5% CO_2_). At various time points (12, 24, 36, and 48 h), cells were harvested from the plate, lysed in 1% Triton-X 100, and then sonicated for 15 sec. The lysates were centrifuged and the supernatants were collected to measure the apo B and TGs concentrations using an apo B ELISA kit and a glycerol-3-phosphate oxidase and phenol 4-aminoantipyrine peroxidase method (GPO-PAP) kit (RANDOX, Antrim, UK).

### 2.7. Animals and Experimental Groups

Fifty 3-week-old male hamsters were purchased from the National Laboratory Animal Center (Taipei, Taiwan). They were housed individually in a controlled environment with 20 ± 2°C temperature, 55 ± 5% humidity, and a 12 h dark-light cycle with the light period at 8 AM to 8 PM. During the first four weeks of the acclimatization period, the animals were fed with chow pellets (AIN-76; Jinlong technology Co., Ltd., Taichung, Taiwan) and water* ad libitum*. They were then randomly divided into one control group and four experimental groups, namely, high-fat and high-cholesterol diet (HFC group) and low- (78 mg/kg, BW/day), medium- (390 mg/kg, BW/day), and high-dose PROBIO S-23 powder (a mixture of NBHK002, NBHK006, and NBHK007) (1950 mg/kg, BW/day) groups. Hamsters in the experimental groups were fed with a basal diet of AIN-76, 12% corn oil, 3% lard, and 0.5% cholesterol 10 days before the experimental period to induce hypercholesterolemia immediately before the experimental period. Different doses of LAB were given orally once a day to the animals in the three PROBIO S-23 groups. PROBIO S-23 powder with high viable counts of LAB (1 × 10^9^–1 × 10^10^ CFU/mL) was produced by freeze-drying (New Bellus Enterprise Co., Ltd., Tainan, Taiwan). Weights of the animals and food intake were recorded. Serum was collected biweekly to measure the concentrations of cholesterol, TG, low-density lipoprotein (LDL), and high-density lipoprotein (HDL). The animals were sacrificed after 10 weeks. Livers were collected to measure the concentrations of cholesterol and TG, as well as the lipid peroxidation index (thiobarbituric acid reactive substance, TBARS). This experimental protocol was approved (number 98002) by the Institutional Animal Care and Use Committee of HungKuang University, Taichung, Taiwan.

### 2.8. Statistical Analysis

Statistical analyses using SPSS 17.0 software (SPSS Inc., Chicago, IL, USA) were performed. Data between groups of animals were compared using one-way analysis of variance. Duncan's multiple range test was performed to determine significant differences. *P* values of < 0.05 were considered statistically significant. Significant differences are indicated by symbols, as shown in the tables and figures.

## 3. Results

### 3.1. Screening the High BSH Activity by LAB Strains

Among the 800 strains screened on plates for BSH activity, only 22 returned positive results (Table 1) with precipitation zones of various sizes (17–24 mm). The eight strains that displayed the largest precipitation zones (NBHK001, NBHK002, NBHK003, NBHK004, NBHK005, NBHK006, NBHK007, and NBHK008) were selected for further study.

**Figure 1:**
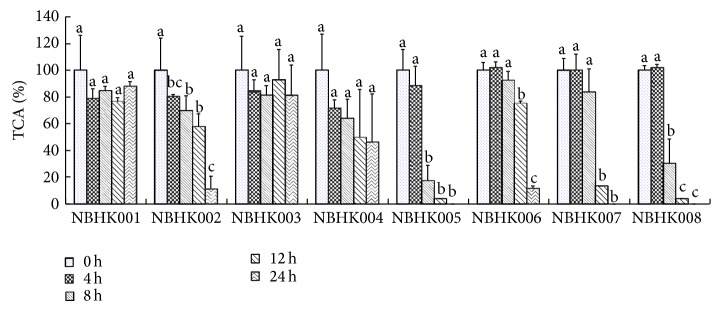
Deconjugation activity of the probiotics. ^a,b,c^Means with different superscript letters within the same culture are significant differences (*P* < 0.05) by Duncan's multiple range test.

**Figure 2:**
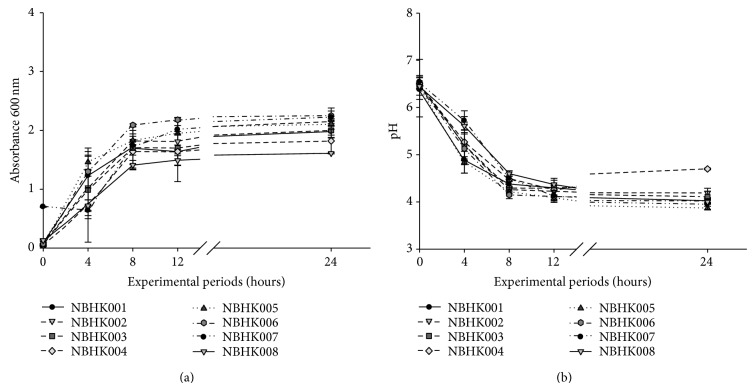
(a) Growth curve of screened probiotics cultured in MRS broth containing TCA. (b) Change in pH value of screened probiotics cultured in MRS broth containing TCA.

When the LAB were grown in MRS broth supplemented with TCA (1 mmol/L each) after 24 h of incubation, strains NBHK002, NBHK005, NBHK006, NBHK007, and NBHK008 showed a notable reduction of TCA during the stationary phase and exhibited stronger deconjugation activity than did the other three strains, NBHK001, NBHK003, and NBHK004 (Figure 1). The growth curve and change in pH of the eight strains during anaerobic incubation at 37°C are shown in Figure 2. Strains NBHK002, NBHK005, NBHK006, and NBHK007 showed a greater reduction in their pH (3.8–4.3) because of greater production of organic acid. Because of the slow growth of NBHK004 and NBHK008, they were excluded from usage in industrial production. Except for NBHK006, a strain of* Bifidobacterium adolescentis*, strains with greater deconjugation ability, namely, NBHK002 and NBHK007, were assayed with the API 50CHL system and 16s rRNA identification to identify the species from Food Industry Research and Development Institute (Hsinchu, Taiwan). The API 50CHL system was used for NBHK002 and NBHK007 strains, demonstrating the highest similarity to* Pediococcus acidilactici* and* Lactobacillus rhamnosus*, respectively. The 16s rRNA identification showed that NBHK002 strain is 98% identical to* Pediococcus acidilactici* and NBHK007 strain has 98.8% sequence identity to* Lactobacillus rhamnosus*.

### 3.2. Cholesterol Concentration in Micelles

**Figure 3:**
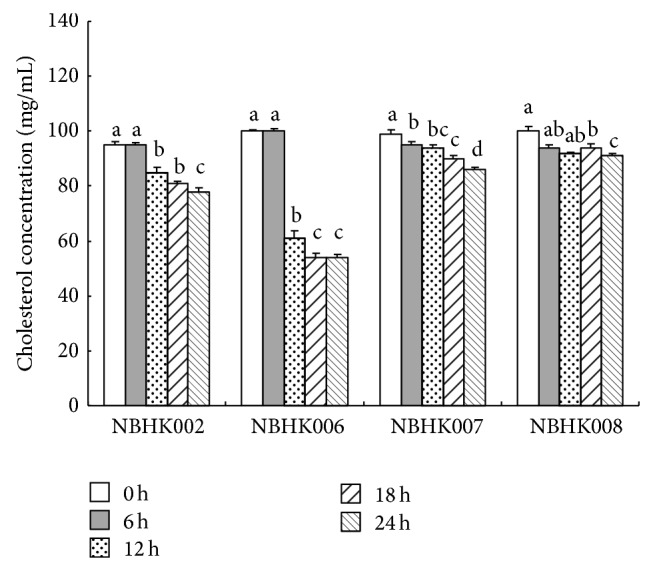
Lowering the micellar solubility of cholesterol by probiotics* in vitro*. ^a,b,c,d^Means with different superscript letters are significant differences (*P* < 0.05) by Duncan's multiple range test.

The effect of the four deconjugative LAB strains on the micellar solubility of cholesterol is shown in Figure 3. The micellar cholesterol concentration was reduced by all LAB strains after 12 h. In particular, NBHK006 produced the greatest reduction in cholesterol concentration (45%) after 18 h.

### 3.3. Inhibition of TG Synthesis and Apo B Secretion by LAB Strains

**Figure 4:**
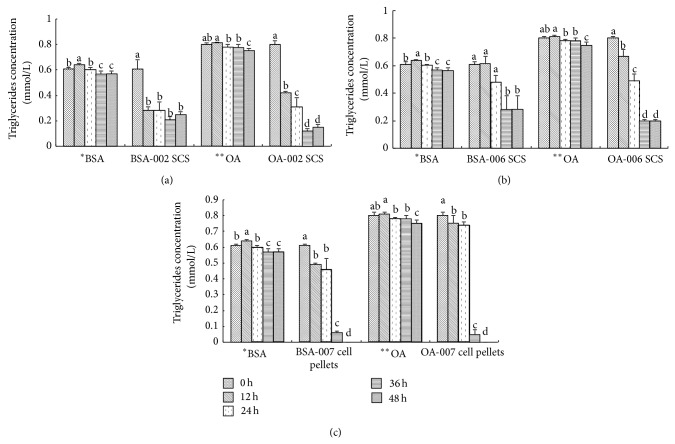
Inhibition of triglycerides in HepG2 cells treated with the spent culture supernatants (SCS) of LAB strains (a) NBHK002, (b) NBHK006, and (c) NBHK007. ^a,b,c,d^Means with different superscript letters within the same culture are significant differences (*P* < 0.05) by Duncan's multiple range test. ^*^BSA: bovine serum albumin; ^**^OA: oleic acid.

**Figure 5:**
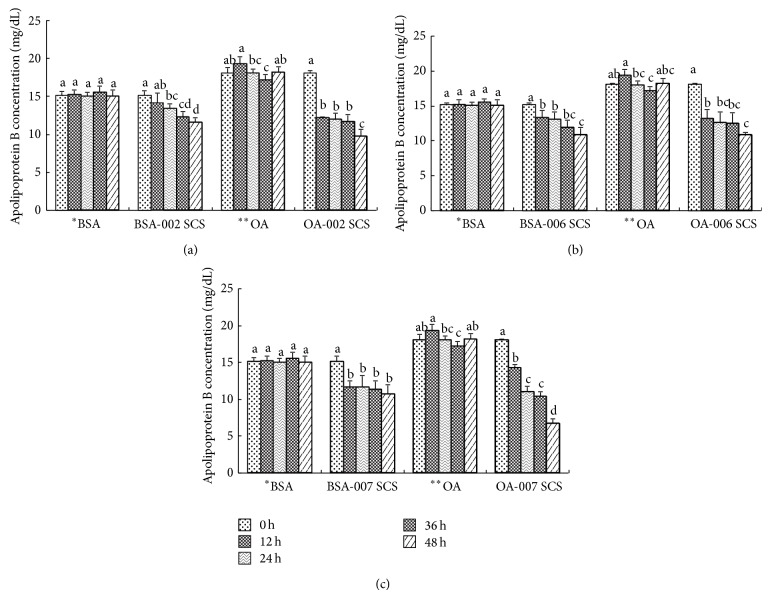
Inhibition of apo B in HepG2 cells which were treated with the suspension of the LAB strains (a) NBHK002, (b) NBHK006, and (c) NBHK007. ^a,b,c,d^Means with different superscript letters within the same culture are significant differences (*P* < 0.05) by Duncan's multiple range test. ^*^BSA: bovine serum albumin; ^**^OA: oleic acid.

Cellular TG synthesis was reduced significantly under lipid-rich conditions in the SCS of the LAB strains (Figure 4) NBHK002, NBHK006, and NBHK007. When NBHK002 TG was added, synthesis decreased by as much as 81% (SCS) in cells incubated with oleate (*P* < 0.05). SCS of NBHK006 reduced TG synthesis in cells by 25–35% in 36–48 h. SCS of NBHK007 inhibited most efficiently, reducing TG synthesis by 77–93% in 36 h and by 100% in 48 h. On the other hand, apo B secretion in OA-treated HepG2 cells decreased over time. Apo B secretion started 12 h after addition of the three LAB strains (Figure 5). Apo B secretion was inhibited by 33%, 38%, and 39% at 24 h in cell suspensions of NBHK002, NBHK006, and NBHK007, respectively.

Because of their high BSH activity and cholesterol-reducing efficacy, LAB strains NBHK002, NBHK006, and NBHK007 were selected and freeze-dried to produce the probiotic formula PROBIO S-23 for further studies* in vivo*.

### 3.4. *In Vivo* Cholesterol-Lowering Effect of PROBIO S-23

**Figure 6:**
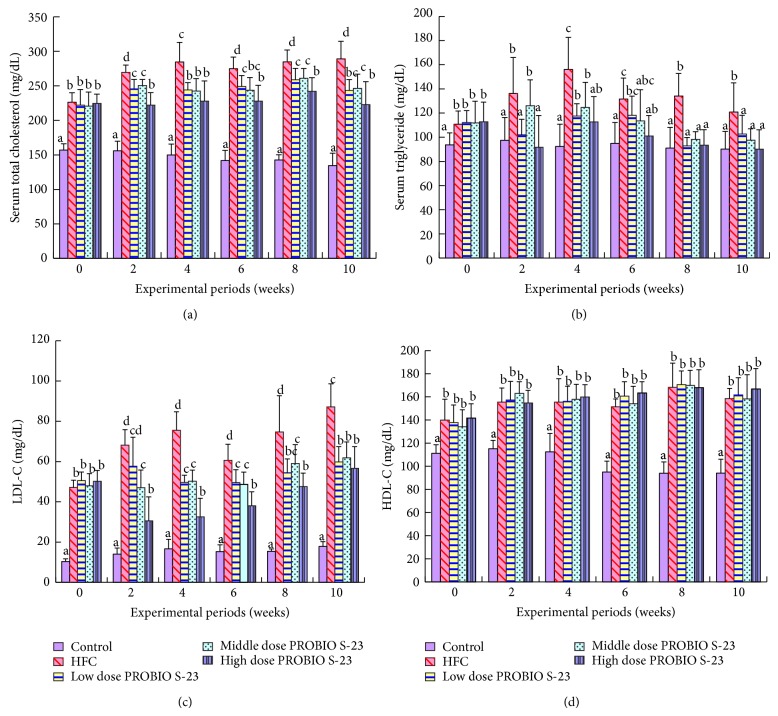
Effects of different concentrations of PROBIO S-23 Complex product on serum (a) total cholesterol, (b) triglyceride, (c) LDL-C, and (d) HDL-C of hamsters fed with high-fat plus high-cholesterol diet during 10 weeks of experimental period.

Throughout the 10-week experimental period, the levels of total cholesterol, TG, and LDL-cholesterol (LDL-C) were significantly lower (*P* < 0.05) in all PROBIO S-23 groups compared with those in the HFC group (Figures 6(a)–6(c)). Compared with the HFC group, hamsters fed with a high dose of PROBIO S-23 showed cholesterol and serum TG levels that were reduced by 22.88% (*P* < 0.05) and 25.53% (*P* < 0.05), respectively, at the end of the experimental period (Figures 6(a) and 6(b)). The high-dose PROBIO S-23 groups showed the greatest reduction of LDL-C levels at weeks 2 and 4 (by 55.23% and 56.96%, resp.), compared with the levels of the HFC group (Figure 6(c)). There was no significant change in HDL-cholesterol (HDL-C) levels between the PROBIO S-23 and HFC groups (Figure 6(d)).

Throughout the 10-week experimental period the levels of total cholesterol, TG, and TBARS in the liver were significantly lower (*P* < 0.05) in all PROBIO S-23 groups compared with those in the HFC group (Table 2). Compared with the HFC group, hamsters fed with a high dose of PROBIO S-23 showed cholesterol, TG, and TBARS levels in the liver that were reduced by 23.9% (*P* < 0.05), 30.33% (*P* < 0.05), and 36.1% (*P* < 0.05), respectively. Similarly, no significant difference was observed in the body weight, food intake, and visceral weight index (*P* < 0.05). No abnormal behavioural changes were observed in the animals in the study groups (data not shown).

## 4. Discussion

The cholesterol-lowering potential of LAB has been discussed in studies for years. In this study, several* in vitro* and* in vivo* experiments were performed to evaluate the ability of LAB to reduce cholesterol levels. Microbial BSH activity in the host results in the reduction of cholesterol levels. Since deconjugated bile acids are less soluble and are less likely to be absorbed from the intestinal lumen than are conjugated bile salts, free bile is more likely to be excreted through the intestinal tract. Therefore, with the help of BSH, deconjugation of bile salts could lead to a reduction of serum cholesterol by reducing cholesterol absorption through the intestinal lumen. This increases the demand for cholesterol for* de novo* synthesis of bile acids to replace their loss through faeces [23]. In our study, the evaluation of BSH activity and growth performance revealed a marked difference in characteristics among the eight strains analysed (Figures 1 and 2). Percentages of TCA were greatly reduced primarily during the stationary phase of the strains. Similar results were observed with certain lactobacilli isolates [24–26]. The study by Nguyen et al. [25], in particular, suggested that BSH activity was greater in the stationary phase compared with that in the exponential phase. Since BSH is predominantly expressed intracellularly, however, no significant correlation was recorded between the BSH activity levels present in resting cells and cell-free extracts (cell lysates) of lactobacilli [27]. Thus, the TCA percentage results may reflect the most BSH enzyme activity.

Strains with greater BSH activity (NBHK002, NBHK005, NBHK006, and NBHK007) showed greater reduction in pH values (3.8–4.3) (Figure 2). Previous studies suggested that the high BSH activity of some lactobacillus species could be partially attributed to the low pH of the medium in the stationary phase [24, 28]. Klaver and van der Meer [29] showed that the degree of deconjugation by* L. acidophilus* RP32 was higher under more acidic conditions than if the pH was maintained at 6.0. They concluded that the removal of cholesterol was due to its coprecipitation with deconjugated bile salts in an acidic environment. The potential positive aspects of probiotic BSH activity have previously been reported, but its possible negative concerns were raised by some investigators [30, 31]. In contrast with this, Kurdi et al. [32] proposed that cholic acid, the main free bile acid produced by BSH activity in the intestine, could accumulate inside the bacterial cells when the bacteria were energised. This bacterial entrapment of free bile acids could contribute to the decreased production of secondary bile acids, which are considered cytotoxic and precarcinogenic. Moreover, 7*α*-dehydroxylase is mainly responsible for this undesirable reaction which has been found in* Clostridium* and* Eubacterium* but not in* Lactobacillus* or* Bifidobacterium*, thus, considerably ruling out the possibility of any harmful effect associated with probiotic strain BSH activity [27, 33].

The absorption of cholesterol in the human body involves emulsification in the stomach, hydrolysis of the ester bond by a specific pancreatic esterase, micellar solubilisation, absorption in the proximal jejunum, reesterification within the intestinal cells, and transportation to the lymph by chylomicrons [34]. Because of the insolubility of cholesterol in water, solubilisation of cholesterol in mixed micelles is a requirement for its efficient absorption [18]. This study simulated the conditions in the human gastrointestinal tract to evaluate the ability of BSH-active strains to remove cholesterol* in vitro*. Our results show that the cholesterol concentration in the mixed micelles decreased from 6 to 12 h upon addition of NBHK002, NBHK006, NBHK007, and NBHK008 (Figure 3). These results suggest that strains were able to remove cholesterol* in vitro* by inhibiting the formation of cholesterol micelles. Noh et al. [35] observed that* L. acidophilus* ATCC 43121 was more resistant to lysis by sonication when grown in the presence of cholesterol micelles and bile salts. They concluded that this resistance may be due to the assimilation of cholesterol into the cellular membrane, resulting in sturdier bacterial cells [35].

Apo B, which is formed in the liver, helps with the stabilisation and transfer of cholesterol and TG and with the removal of cholesterol in the liver and the outer tissues. In a previous study xanthohumol was evaluated for its ability to inhibit TG synthesis and regulate apo B secretion in HepG2 cells [22]. TG availability is a determining factor in apo B secretion regulation. In the present study we used HepG2 cells to examine the probiotics effect on apo B and TG secretion. The results show that the strains NBHK002, NBHK006, and NBHK007 had greater ability to reduce both apo B and TG secretion in HepG2 cells.

Developing the PROBIO S23 formulation in dried form using the three probiotic strains in the animal model and establishing increasing faecal count with* Lactobacillus* and* Bifidobacterium* (8.49–10.21 log CFU/g). These evidences suggested that the PROBIO S-23 could colonize gastrointestinal epithelia and had symbiotic action among the three probiotic strains. In the animal model serum TG, total cholesterol and LDL levels decreased after PROBIO S-23 administration at low, medium, or high dosages (Figure 6), without affecting the structure and relative weight of the liver. No significant improvement in HDL-C levels was observed. Oral administration of probiotics has been shown to significantly reduce cholesterol levels by as much as 22 to 33% [9] or prevent elevated cholesterol levels in mice that have been fed with a fat-enriched diet [26]. However, deconjugation of bile salts only partly explains the hypocholesterolemic effect of probiotics. Other mechanisms may also contribute to this effect. It has been suggested that the assimilation of cholesterol during probiotic LAB growth and cholesterol binding to their cellular membrane results in lowered availability of cholesterol for absorption, leading to reduced serum cholesterol in the host [19, 29]. Probiotics may also produce SCFAs such as butyrate, which have been studied for liver cholesterol synthesis inhibition in blocking HMG-CoA reductase activity, which is a rate-limiting enzyme and is involved in endogenous cholesterol production, as well as decreasing the transformation of primary to secondary bile acids as a result of colonic acidification [23, 36]. Thus, BSH-active and TG-lowering strains in PROBIO S-23 might improve the lipid profile in the animals in our study through deconjugation of bile salts and other mechanisms.

The hypocholesterolemic mechanism effect of probiotics might be strain-specific.* L. fermentum* normally adheres to epithelial cells in the human gastrointestinal tract and promotes the survival of healthy intestinal microflora [37]. In our study selected strains in PROBIO S-23 were* L. rhamnosus *(NBHK007),* B. adolescentis* (NBHK006), and* P. acidilactici* (NBHK002). However,* L. rhamnosus* (NBHK007) reduced TG synthesis by 77–100% and inhibited apo B secretion by 39% in cell culture, resulting in the most efficient reduction among the strains.* L. fermentum* isolated from fermented milk has been found to exhibit acid and bile tolerance and to reduce serum TG and LDL-C levels. However, it did not increase HDL-C levels in mice [6]. Lee et al. [38] evaluated the hypocholesterolemic effect of* Bifidobacterium *spp. isolated from faecal samples of healthy Koreans and found that* B. adolescentis* had the ability to lower cholesterol in experiments conducted* in vitro* or* in vivo*. Strains of the same species have different effects on the cholesterol level. Doumandji et al. conducted an* in vitro* experiment by combining* B. adolescentis* and spirulina and observed significant degradation in total cholesterol after 72 h. Screening of* P. pentosaceus* in traditional Thai fermented food, wine, and beer was performed to determine its ability to produce exopolysaccharides (EPs) [39–41]. Several studies indicated the beneficial effects of EPs on human health, including cholesterol-lowering effect. For example, the EP produced by* Lactobacillus kefiranofaciens*, kefiran, was reported to reduce serum cholesterol levels as well as suppress the increase in blood pressure in SHRSP/Hos rats consuming excessive amounts of cholesterol [42]. Pigeon et al. also suggested that cholesterol removal by* Lactobacillus delbrueckii* and* Streptococcus thermophilus* strains was due to the binding of free bile acids to their cell membranes through extracellular polysaccharides [43]. Similarly, results of our study indicate that PROBIO S-23 is a safe, multistrain probiotic product with the potential to reduce serum cholesterol and TG levels and their levels in the liver. Tok and Aslim [44] suggest that the EPS produced by the bacteria interacted with the cholesterol in the medium and bound it in a manner like a dietary fiber. They reported that immobilized cells were much effective in cholesterol adsorption than free cells.

In conclusion, the probiotic strains isolated and characterized in this study have great potential as possible therapy for reducing cholesterol levels. The cholesterol-lowering effects of PROBIO S-23 presented may be partially ascribed to BSH activity* in vitro*. This product exerted a significant hypocholesterolemic effect on hamsters fed with an HFC diet. PROBIO S-23 consumption as a probiotic dietary supplement might be useful in reducing total cholesterol and TG levels in the serum and liver for humans. PROBIO S-23 appears to be safe for its potential use in hypercholesterolemia control.

## Figures and Tables

**Table 1:** Screening of the bile salt hydrolase (BSH) activity of probiotics using the plate assay method.

Strain	MRS agar	MRS agar (0.5% TDCA)	MRS agar (0.5% TCA)
	Precipitation zone diameter (mm)^a^		
NBHK001	10 ± 1	—^b^	19 ± 4
NBHK002	10 ± 1	19 ± 4	16 ± 2
NBHK003	11 ± 1	12 ± 1	18 ± 3
NBHK004	10 ± 1	23 ± 4	15 ± 4
NBHK005	11 ± 1	14 ± 0	19 ± 2
NBHK006	9 ± 1	14 ± 3	20 ± 4
NBHK007	13 ± 3	24 ± 4	18 ± 2
NBHK008	9 ± 1	20 ± 4	20 ± 1
LGA-01	11 ± 1	—	16 ± 1
LRE-01	11 ± 0	17 ± 0	—
Bgal-1	11 ± 1	19 ± 0	12 ± 0
TS 111	11 ± 0	16 ± 0	13 ± 0
TS 159	11 ± 0	15 ± 0	14 ± 0
TS 6	11 ± 0	14 ± 0	11 ± 0
TS 17	12 ± 0	17 ± 0	16 ± 0
TS 18	11 ± 1	17 ± 0	16 ± 1
TS 26	11 ± 1	17 ± 0	16 ± 0
TS 29	11 ± 1	16 ± 0	18 ± 4
TS 32	13 ± 0	19 ± 0	—
RY11-M2	10 ± 1	16 ± 0	—
MY22-M1	9 ± 1	13 ± 4	—
RY11-R1	14 ± 6	16 ± 3	—

^a^Diameter of precipitation zone included 6 mm diameter of the sterile filter disk.

^b^Not detected.

**Table 2:** Effects of liver lipid profiles of hamsters fed with high fat plus high cholesterol diet after 10 weeks of treatments with different concentrations of PROBIOS-23 Complex product.

Groups	Cholesterol (mg/g)	Triglyceride (mg/g)	TBARS (MDA uM)
Control	6.32 ± 1.43^a^	27.57 ± 3.33^a^	7.73 ± 0.98^b^
HFC	48.07 ± 5.76^c^	47.24 ± 4.79^d^	9.61 ± 1.33^c^
Low dose PROBIO S-23	36.58 ± 8.19^b^	38.39 ± 4.54^c^	7.15 ± 0.36^ab^
Middle dose PROBIO S-23	33.58 ± 6.62^b^	35.20 ± 3.33^bc^	7.80 ± 1.15^b^
High dose PROBIO S-23	34.24 ± 7.04^b^	32.91 ± 5.99^b^	6.14 ± 1.51^a^

Data are expressed as means ± SD (*n* = 8–10).

^a,b,c,d^Values in the same column with different superscripts are significantly different (*P* < 0.05).
